# Acute Knee Injury-Related TikTok Videos Are Frequently Inaccurate, Incomplete, and Created by Nonphysicians

**DOI:** 10.1016/j.asmr.2025.101195

**Published:** 2025-05-31

**Authors:** Levi M. Travis, Jacob Jahn, Natalie K. Blanc, Lee Kaplan

**Affiliations:** Department of Orthopedic Surgery, University of Miami Miller School of Medicine, Miami, Florida, U.S.A.

## Abstract

**Purpose:**

To evaluate TikTok videos related to knee injuries, examining the accuracy and sources of the content, the category of information provided, the reach of the videos, and the capability of the videos to cause harm.

**Methods:**

On September 1, 2024, TikTok was queried using layperson’s terms for acute knee injuries (e.g., “ACL tear”) to identify popular hashtags. The top 10 videos per hashtag and 5 videos per search term (e.g., “knee pop”) by view count were included if they related to the specified knee injury, surgery, or recovery process. Videos with fewer than 1,000 views were excluded. Metrics such as number of likes, number of views, number of comments, creator demographic characteristics, and video content type were collected, and videos were evaluated for quality using the DISCERN scoring system.

**Results:**

A total of 234 TikTok videos related to knee injuries were analyzed, averaging 699,235 views per video (median, 138,500 views). DISCERN analysis revealed that 41% of videos were rated as poor whereas 59% were satisfactory. Videos featuring medical recommendations had significantly higher engagement scores (mean, 5.16; 95% confidence interval [CI], 3.49-6.83; *P* = .001) and longer durations (mean, 53.38 seconds; 95% CI, 44.47-62.28 seconds; *P* = .002) than those without recommendations (mean score, 3.18 [95% CI, 2.84-3.52]; mean duration, 38.10 seconds [95% CI, 33.18-43.01 seconds]). Satisfactory videos outperformed poor-quality videos across DISCERN metrics, with clearer aims (mean, 3.91 vs 2.72; *P* < .001), greater relevance (mean, 3.20 vs 2.09; *P* < .001), and more balanced information (mean, 1.60 vs 1.00; *P* < .001). Physicians created 24.4% of the videos, which generally scored higher according to the DISCERN criteria than videos created by non–health care–related professionals.

**Conclusions:**

Videos created by health care professionals, particularly physicians, scored higher in terms of educational quality but accounted for a small proportion of total content. In contrast, nonphysician creators frequently provided inaccurate or incomplete information. Despite this, videos with medical recommendations achieved higher engagement.

**Clinical Relevance:**

There is a predominance of nonphysician creators disseminating inaccurate medical information, underscoring the need for orthopaedic surgeons to engage in digital health education to provide reliable content. This study provides insights into the digital content patients may be consuming regarding their medical conditions.

The inundation of health information through the internet poses a challenge to physicians in guiding their patients’ decision making. The internet has increased accessibility to learn about health conditions, but with large amounts of content come some inaccurate, dangerous sources that may pose risks to patient health.^1^ Misinformation can lead to misunderstandings about medical conditions, unrealistic treatment expectations, or patients following suboptimal recovery plans with no medical support. Social media has become a source of information for many in recent years, with 4.62 billion users by the end of 2023, and TikTok (ByteDance) has recently emerged as a popular platform, engaging 33% of social media users.^2^ This reliance on social media for everyday information, including personal health care information, indicates that there is a need to investigate the quality of information posted. It is essential that TikTok has accurate and nonharmful advice to support informed health decisions by patients.^2^ Physicians, health care providers, nontraditional health professionals, and patients can contribute to TikTok, covering a wide range of information from surgical simulations to personal surgery recovery experiences. Although these videos can offer helpful insights, is there significant variability in content quality?

A direct relationship between the quality of health information and patient outcomes has been seen in orthopaedic research given that inadequate health literacy may correlate with subpar outcomes and decision making.^3^ Obana et al.^3^ found that patients turn to the internet to learn about diagnoses, treatment, and recovery more frequently than details about the cost or specific details of the procedure. A study analyzing content on TikTok and Instagram (Meta Platforms) found inaccuracies of information regarding Achilles tendon tears, anterior cruciate ligament (ACL) tears, and meniscal injuries and suggested that increased involvement from medical professionals could improve the quality of posted content.^4^ Furthermore, health literacy has been shown to influence patient expectations and satisfaction before and after knee surgical procedures,^5^ indicating a need to further analyze educational disparities in online health information.

Despite the popularity of TikTok with young individuals, limited research has assessed the accuracy and educational quality of its content regarding knee injuries. One study found that 81.68% of TikTok users in China were younger than 35 years and 32.5% of TikTok users in the United States were younger than 19 years.^6^ Because the knee is the most commonly injured joint in adolescent athletes and given that adolescents frequently consume content on TikTok, evaluating the educational quality of acute knee injury videos is essential to work toward mitigating potential harm to patients.^7^ The purpose of this study was to evaluate TikTok videos related to knee injuries, examining the accuracy and sources of the content, the category of information provided, the reach of the videos, and the capability of the videos to cause harm. We hypothesized that TikTok would be a significant source of health information on knee injuries but the quality of content would vary widely and tend to be substandard; videos created by physicians would show higher educational quality compared with those created by nonphysicians; and videos featuring medical recommendations would receive higher engagement from viewers.

## Methods

### TikTok Query

TikTok was queried on September 1, 2024, and all video identifiers were collected on that date. Video and creator metrics were collected between September 3 and 13, 2024. Video querying was performed using a new TikTok account without prior metadata or search history. Inclusion criteria consisted of videos with content directly related to the search terms or hashtags and ranked in the top 10 videos by view count by hashtag query or in the top 5 videos by view count by search term. Videos unrelated to the search term, videos with fewer than 1,000 views, and duplicate videos were excluded from data collection.

For query term development, top hashtags associated with each knee pathology, surgical procedure, and recovery were first identified: For example, TikTok was queried for “ACL tear”; the top 10 videos, sorted by like count, were reviewed; and the most common hashtags (e.g., #acltear, #tornacl, #aclinjury, #aclsurgery, #aclreconstruction, #aclrehab, and #aclrecovery) were included. The same protocol was applied regarding medial collateral ligament tear, lateral collateral ligament tear, and meniscal tear. Knee surgery and knee arthroscopy were also searched on TikTok, which identified #kneesurgery, #kneesurgeryrecovery, #kneearthroscopy, and #arthroscopy as the most common hashtags. Other search terms included were “multiligament knee,” “bucket handle tear,” “knee pop,” “knee tear,” “knee swelling,” “knee edema,” “knee instability,” and “unstable knee.” These hashtags and search terms were then used for the aforementioned queries.

### Data Collection

For each video, video metrics including numbers of likes, views, shares, comments, and favorites were collected. Engagement was calculated by summing the video's likes, shares, comments, and favorites, dividing by the views and multiplying by 100. Creator information including sex, profession (physician, health care nonphysician, nontraditional health care, or non–health care), degree (M.D., D.O., alternative doctoral degree, or undisclosed), organization (non-organization, profit, or nonprofit), traditional versus alternative medicine (any treatment used instead of standard medical care including chiropractic treatment and acupuncture), total number of followers, total number of likes, and total number of views was collected. Video length, agreeability of comments (top 3 comments by like count overall agreeable vs disagreeable with video content), and content type (disease/symptom information, exercises, treatment, recovery, and diagnosis) were recorded. Symptoms described were categorized as pain and swelling (including fluid) or tenderness; change in range of motion; locking, popping, clicking, or instability; or a combination of pain/swelling and locking/popping. Exercises suggested were categorized as flexion and extension (includes body weight exercises), weight lifting, banded exercises or lateral movements, or balance/stretching. Treatments suggested or described were categorized as conservative, surgery, or injections/other intermediate treatment. It was noted whether medical recommendations were made and subjectively whether the content was considered harmful or nonharmful and poor or satisfactory. The content type was also recorded (educational, anecdotal, or commercial).

### DISCERN Scoring

DISCERN scoring was adapted to evaluate the reliability and information quality of video content and has been used in previous studies analyzing social media health information.^8^ The scoring system involves 16 items assessing both the reliability of information and the quality of treatment-related content. Each video was given 1 to 5 points for each of 16 categories. An excellent score is 63 to 80 points; good, 51 to 62 points; fair, 39 to 50 points; poor, 27 to 38 points; and very poor, 16 to 26 points.

## Results

### Means and Frequencies

A total of 234 TikTok videos focused on knee injuries were analyzed. On average, videos accumulated 699,235.69 views (median, 138,500 views), with 27,750.33 likes (median, 3,083 likes), 1,229 shares, and 233 comments (Table 1). Videos received a mean of 3,556.72 favorites (median, 2,890 favorites). Creators had a mean 565,970 followers and 12,160,214.24 likes (Table 1). The average video length was 42.76 seconds (standard deviation, 34.25 seconds). The mean overall DISCERN score was 2.07 (standard deviation, 0.904), with videos scoring poorly in terms of balance, sourcing, and treatment risk identification (Table 2).

**Table 1:** Descriptive Data for Video and Creator Statistics

	Video and Creator Statistics								
	No. of Views	No. of Likes	No. of Shares	No. of Comments	No. of Favorites	Engagement	Total No. of Followers	Total No. of Likes	Video Length
n	234	234	234	234	234	234.00	233	233	234.00
Mean	699,236	27,750	1,229	233	3,557	3.80	565,970	12,160,214	42.76 s
Median	138,500	3,083	201	59	634	2.89	64,600	1,300,000	37.00 s
SD	1,517,784	91,971	3,164	618	8,544	4.41	1,630,187	25,556,079	34.25 s
Percentiles									
25th	38,500	724	52	16	143	1.87	16,200	262,350	19.00 s
50th	138,500	3,083	201	59	634	2.89	64,600	1,300,000	37.00 s
75th	513,525	12,950	981	164	2,328	4.57	391,100	12,300,000	58.00 s

NOTE. Summary statistics including video engagement metrics (numbers of views, likes, shares, comments, favorites, and total followers) and video length are presented for 234 TikTok videos related to knee injuries.

SD, standard deviation.

**Table 2:** Descriptive Data for DISCERN Statistics

	DISCERN Statistics															
	Aims Clear	Achieve Aims	Relevant	Sources	Source Time	Balanced and Unbiased	Additional Sources	Areas of Uncertainty	Tx Mechanism	Tx Benefits	Tx Risks	No Tx Risk	Tx QOL	≥2 Tx	Shared Decision Making	Overall Rating
n	234	226	234	234	234	234	233	234	147	146	147	147	147	147	213	232
Mean	3.42	3.20	2.74	1.76	1.09	1.39	1.07	1.12	2.61	2.00	1.19	1.14	1.30	1.67	1.25	2.07
Median	3.00	3.00	3.00	2.00	1.00	1.00	1.00	1.00	3.00	1.00	1.00	1.00	1.00	1.00	1.00	2.00
SD	1.054	0.971	0.919	0.770	0.459	0.686	0.463	0.524	1.168	1.186	0.553	0.597	1.069	1.106	0.911	0.904
Percentiles																
25th	3	3	2	1	1	1	1	1	2	1	1	1	1	1	1	1
50th	3	3	3	2	1	1	1	1	3	1	1	1	1	1	1	2
75th	4	4	3	2	1	2	1	1	3	3	1	1	1	2	1	3
90th	5	5	4	3	1	2	1	1	4	4	2	1	2	3	1	3

NOTE. Summary statistics for DISCERN quality ratings are presented for 234 TikTok videos related to knee injuries. DISCERN ratings assess video clarity, sourcing, balance, treatment information, and overall quality.

QOL, quality of life; SD, standard deviation; Tx, treatment.

Most creators were male (86.1%), with 24% being physicians, 39.9% being other professionals, 6.9% being nontraditional providers, and 29.2% having no health care background (Table 3). Of the creators, 21.6% held an M.D. degree, 2.9% had a D.O. degree, and 20.5% possessed other doctoral degrees, whereas 55.0% of creators did not disclose their educational background. Most videos (67.1%) were produced by individuals not affiliated with an organization, whereas 31.6% came from profit organizations and only 1.3% came from nonprofit organizations. Most content adhered to traditional medical perspectives (86.3%), with 13.7% representing alternative approaches (Table 3).

**Table 3:** Demographic and Professional Characteristics and Video Content

	n	%
Creator sex		
Male	199	86.1
Female	31	13.4
Creator profession		
Physician	56	24.0
Health care nonphysician	93	39.9
Nontraditional health care	16	6.9
Non–health care	68	29.2
Physician		
No	177	75.6
Yes	57	24.4
Creator degree		
M.D.	37	21.6
D.O.	5	2.9
Alternative doctoral degree	35	20.5
Undisclosed	94	55.0
Organization type		
Non-organization	157	67.1
Profit	74	31.6
Nonprofit	3	1.3
Traditional vs alternative medicine		
Traditional	138	86.3
Alternative	22	13.7
Top 3 comments		
Agreeable	204	91.9
Disagreeable	18	8.1
Content type		
Disease/symptom information	53	22.6
Exercises	56	23.9
Treatment	62	26.5
Recovery	51	21.8
Diagnosis	12	5.1
Symptoms described		
Pain, swelling, or tenderness	35	48.6
Change in ROM	9	12.5
Locking, popping, clicking, or instability	21	29.2
Pain, swelling, or tenderness and instability	7	9.7
Exercises described		
Flexion/extension	59	69.4
Other weight-lifting exercises	7	8.2
Banded exercises or lateral movements	13	15.3
Balance/stretching	6	7.1
Treatment described		
Conservative	91	57.2
Surgery	59	37.1
Injections/intermediate	9	5.7
Medical recommendations made		
No	161	69.1
Yes	72	30.9
Video context		
Educational	159	67.9
Anecdotal	70	29.9
Commercial	5	2.1
Subjective rating		
Poor	96	41.0
Satisfactory	138	59.0
Subjectively harmful		
No	218	93.2
Yes	16	6.8

NOTE. Demographic and professional characteristics of video creators, content themes, and engagement metrics are summarized for TikTok videos related to knee injuries. Variables include creator sex, profession, educational background, and organizational affiliation. Additionally, video content type, symptoms described, exercises and treatments featured, medical recommendations, and subjective ratings (including harmful content) are reported. Percentages reflect the proportion of total videos for each category.

ROM, range of motion.

Most videos (91.9%) featured primarily agreeable comments whereas 8.1% contained disagreeable comments. Content was divided into several categories, with 22.6% of videos addressing disease or symptom information, 23.9% demonstrating exercises, 26.5% focusing on treatments, 21.8% covering recovery strategies, and 5.1% discussing diagnoses. Symptom descriptions included pain, swelling, or tenderness in 48.6% of videos; changes in range of motion in 12.5%; locking, popping, clicking, or instability in 29.2%; and combined pain and instability in 9.7% (Table 3). Exercise-related content primarily featured flexion/extension movements (69.4%), whereas 15.3% of videos included banded exercises or lateral movements, 8.2% showed other weight-lifting exercises, and 7.1% focused on balance and stretching routines. Conservative treatment approaches were the most frequently discussed (57.2%), followed by surgical interventions (37.1%) and injection-based or intermediate treatments (5.7%) (Table 3). Medical recommendations appeared in 30.9% of videos. Most content was educational (67.9%), with 29.9% being anecdotal and 2.1% promoting commercial products. On subjective assessment, 41.0% of videos were rated as poor whereas 59.0% were satisfactory. In addition, 6.8% of the videos were deemed harmful.

### Comparisons of Means

**Fig 1:**
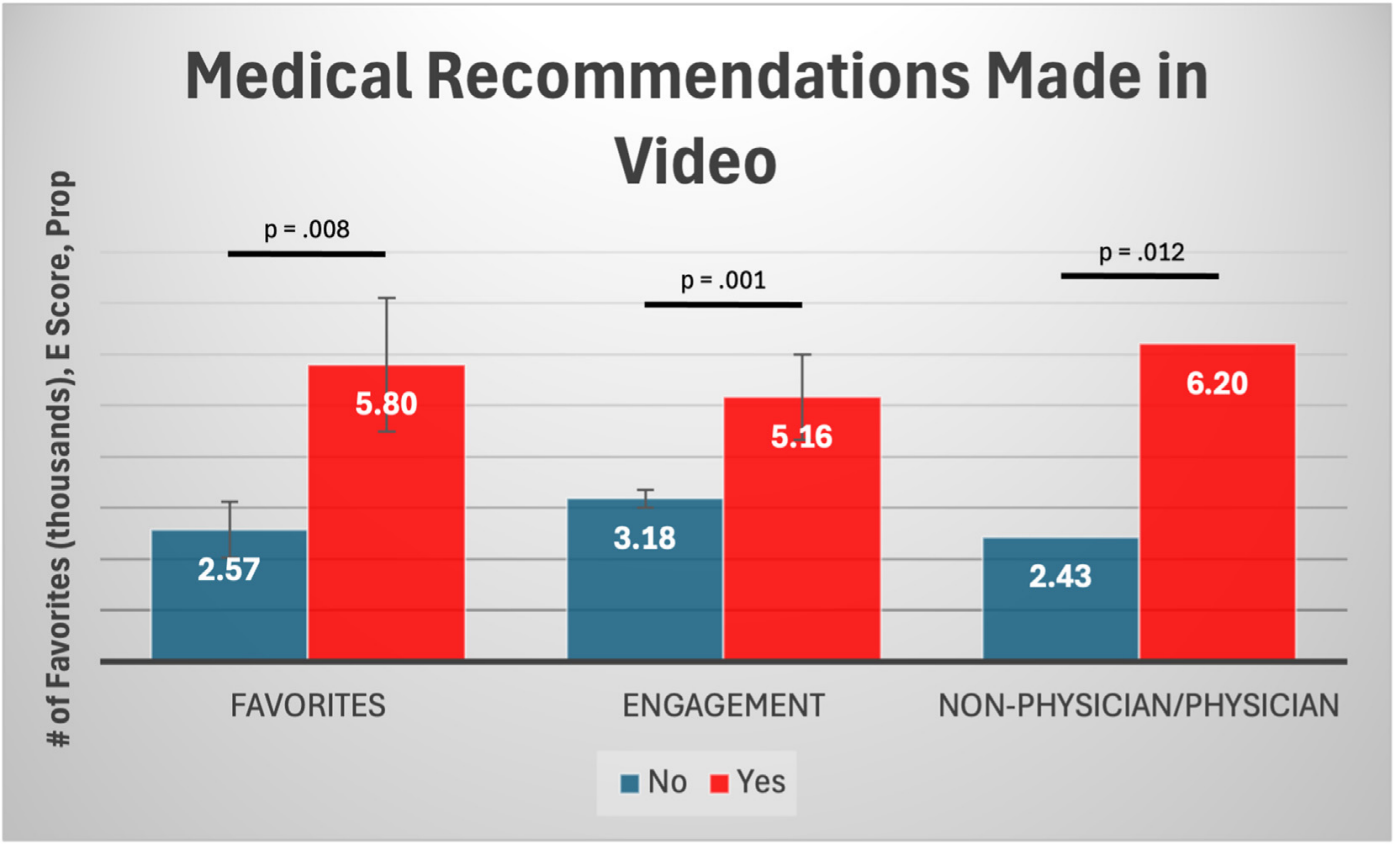
Differences in number of favorites (in thousands), overall engagement, and creator type (physician vs nonphysician) between TikTok videos that included medical recommendations (red) and those that did not (blue). The proportion of nonphysicians to physicians was higher in videos that made medical recommendations. Error bars represent standard errors. Statistically significant associations are represented by *P* < .05.

**Fig 2:**
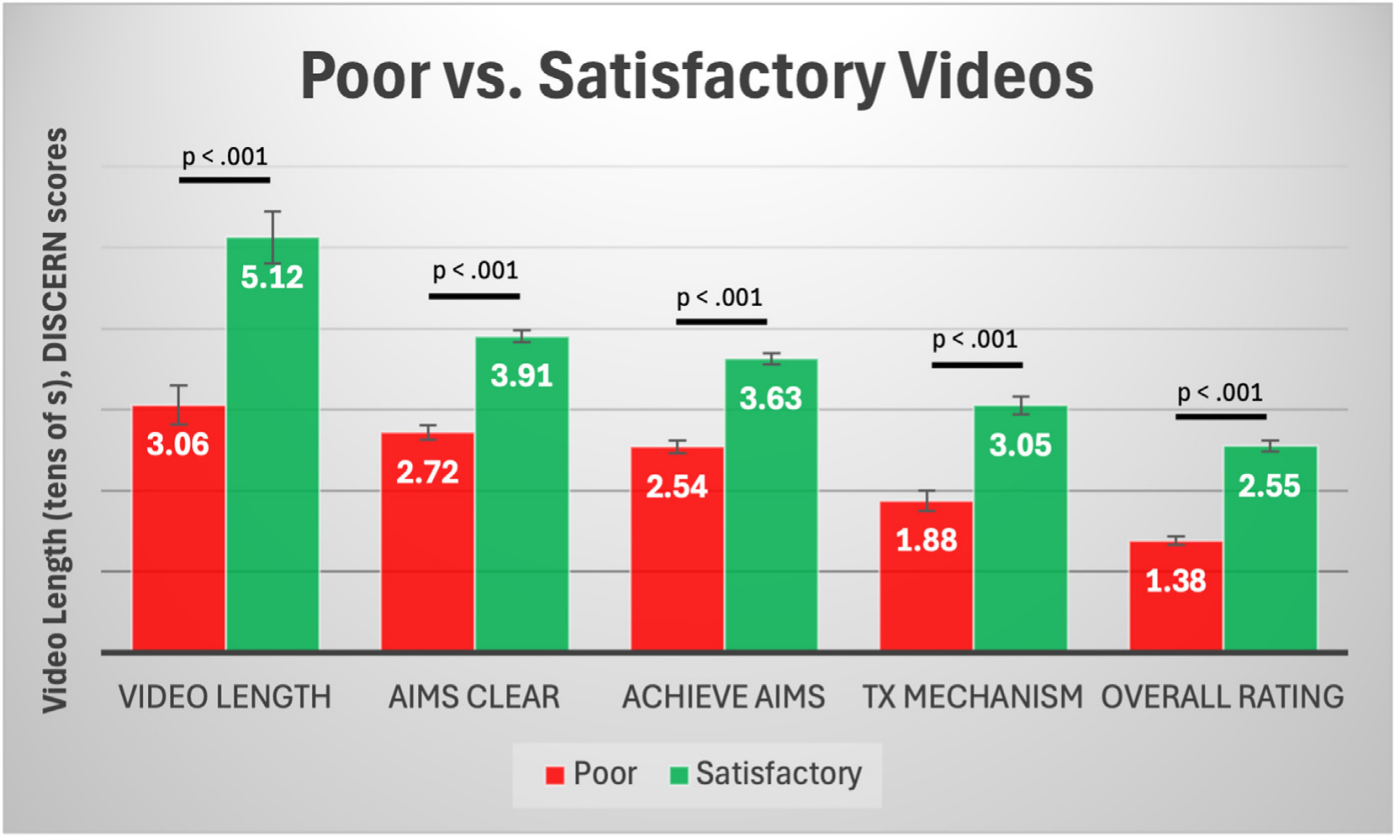
Comparison of video length and DISCERN criteria scores between poor-quality (red) and satisfactory (green) TikTok videos related to knee injuries. Error bars represent standard errors. Statistically significant associations are represented by *P* < .05.

Physician creators had lower engagement scores than nonphysician creators (mean, 1.97 vs 4.38; *P* < .001). Videos with medical recommendations had higher engagement scores (mean, 5.16 vs 3.18; *P* = .001), were longer (mean, 53.38 seconds vs 38.10 seconds; *P* = .002), and scored higher in terms of clarity (mean, 3.68 vs 3.30; *P* = .012) and sources cited (mean, 1.96 vs 1.68; *P* = .011) than those without recommendations (Fig 1). Satisfactory videos scored higher than poor-quality videos in terms of clarity of aims (mean, 3.91 vs 2.72; *P* < .001), aim achievement (mean, 3.63 vs 2.54; *P* < .001), and relevance (mean, 3.20 vs 2.09; *P* < .001) (Fig 2). These videos also scored higher in terms of sources (mean, 2.08 vs 1.31; *P* < .001) and balanced information (mean, 1.60 vs 1.08; *P* < .001) compared with poor-quality videos.

Videos rated as satisfactory were significantly longer (mean, 51.24 seconds; 95% confidence interval [CI], 44.88-57.60 seconds) than poor-quality videos (mean, 30.57 seconds; 95% CI, 25.77-35.38 seconds; *P* < .001) (Fig 2). They also scored higher in terms of treatment clarity and treatment mechanism (mean, 3.05 vs 1.88; *P* < .001) and explaining multiple treatment options (≥2 treatments) (mean, 1.89 vs 1.30; *P* = .002). Subjectively harmful videos did not significantly differ from nonharmful videos across most engagement metrics. However, subjectively harmful videos scored lower in terms of achieving aims (mean, 2.60 [95% CI, 2.10-3.10] vs 3.24 [95% CI, 3.11-3.37]; *P* = .013) and relevance (mean, 2.25 [95% CI, 1.84-2.66] vs 2.78 [95% CI, 2.66-2.90]; *P* = .026). Additionally, harmful videos received lower overall ratings (mean, 1.44; 95% CI, 1.00-1.87; *P* = .004) than nonharmful videos (mean, 2.12; 95% CI, 2.00-2.24). Profit organizations tended to have greater total numbers of followers (mean, 1,203,432; *P* < .001) and total numbers of likes (mean, 21,434,299; *P* = .001) than non-organizational creators. Videos from profit organizations also showed significantly higher engagement scores (mean, 3.45; 95% CI, 2.98-3.93) compared with videos from nonprofit creators (mean, 11.70; 95% CI, –32.64 to 56.04; *P* = .006).

**Fig 3:**
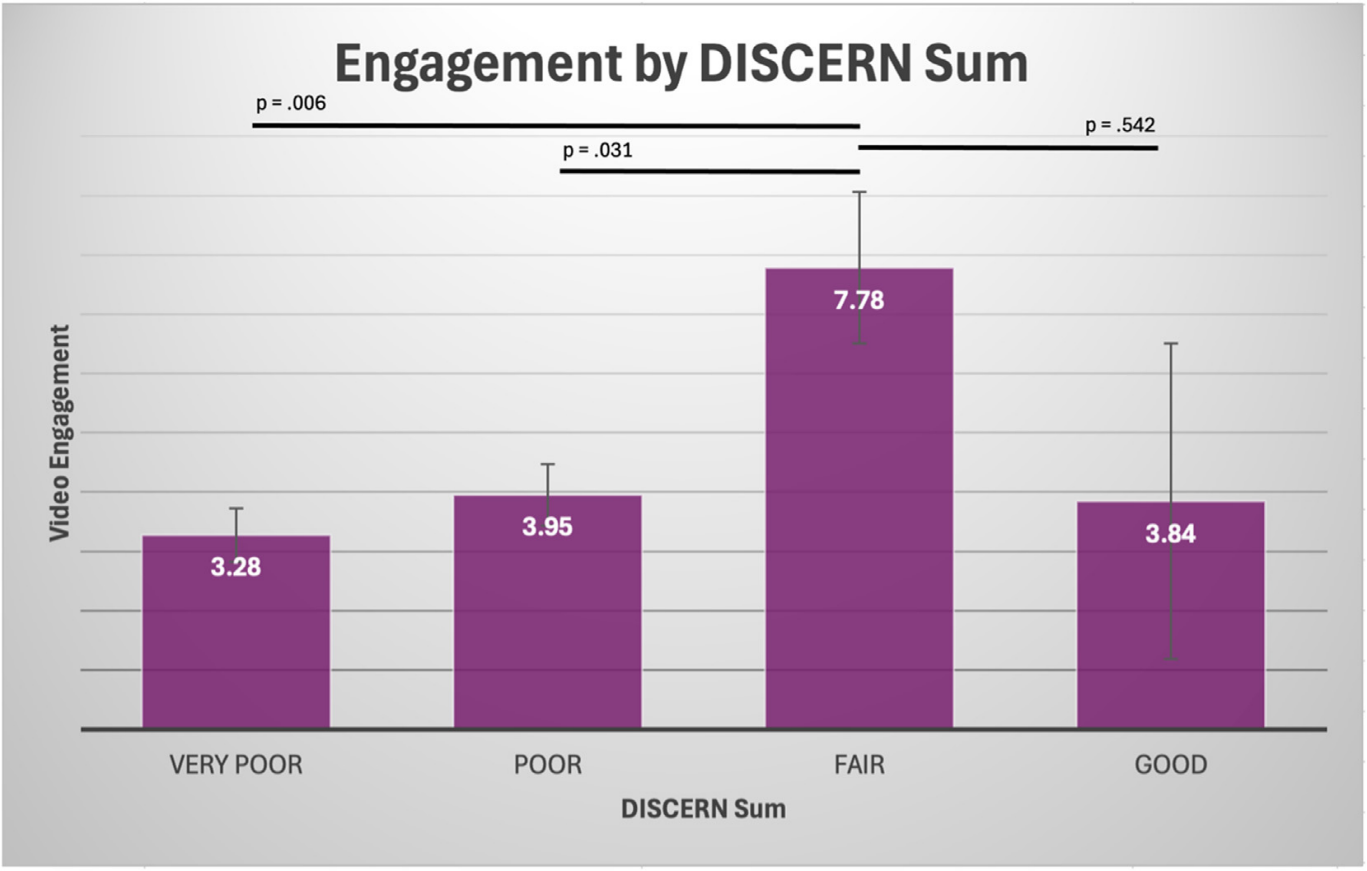
Relationship between video engagement and DISCERN quality ratings for knee injury–related TikTok videos. Engagement was significantly higher for videos rated as fair (DISCERN summed score, 39-50 points) compared with very poor (16-26 points, *P* = .006) and poor (27-38 points, *P* = .031), but there was no significant difference between videos rated fair and good (51-62 points, *P* = .542). Error bars represent standard errors. Statistically significant associations are represented by *P* < .05.

By use of the previously defined ranges for classifying summed (total) DISCERN scores, videos receiving a fair score had significantly more engagement than those receiving poor (*P* = .006) and very poor (*P* = .031) scores but showed no difference compared with videos receiving a good score (*P* = .542). Only 3 videos were classified as good (Fig 3).

### Regressions

Overall rating had a significant negative relationship with video views (*P* = .030, β = –240,138.4, standard error [SE] = 110,001.6), video likes (*P* = .031, β = –14,447.19, SE = 6,666.38), and video comments (*P* = .021, β = –104.22, SE = 44.73). In addition, overall rating was positively related to video length (*P* < .0001, β = 12.89, SE = 2.35).

Video length had a significant positive relationship with engagement (*P* = .0003, β = 0.030, SE = 0.008) and the following DISCERN categories (Table 4): aims clear, achieve aims, relevance, sources, balanced and unbiased, treatment mechanism, treatment benefits, 2 or more treatments, and overall rating (*P* < .0001, β = 0.009, SE = .0016).

**Table 4:** Association of Video Length With DISCERN Scores

	Association With Video Length		
	β	SE	*P* Value
Aims clear	0.0075	0.002	<.01
Achieve aims	0.0068	0.002	<.01
Relevant	0.0068	0.002	<.01
Sources	0.0035	0.002	.02
Balanced and unbiased	0.0040	0.001	<.01
Treatment mechanism	0.0067	0.003	<.01
Treatment benefits	0.0058	0.003	.03
≥2 Treatments	0.0065	0.002	<.01
DISCERN overall rating	0.0090	0.002	<.01

NOTE. The results of a linear regression analysis examining the relationship between video length (in minutes) and various DISCERN quality criteria are presented for TikTok videos on knee injuries. The β coefficients represent the effect size for each criterion, with *P* values indicating statistical significance. Statistically significant associations are represented by *P* < .05.

SE, standard error.

### Cross-tabulation Findings

Educational videos were more likely to receive a satisfactory rating (73.0%) compared with anecdotal videos [72.9% rated poor, χ^2^(2) = 42.164, *P* < .001]. Medical recommendations were significantly associated with physician status [χ^2^(1) = 6.305, *P* = .012], with nonphysician creators making medical recommendations more frequently (35.2%) than physicians (17.5%). Physicians were more likely to produce videos about treatments (41.1%) and diagnosis (10.5%), whereas nonphysicians predominantly created videos about exercises (31.1%) and recovery (26.0%) [χ^2^(4) = 39.553, *P* < .001]. Conservative treatments were more commonly recommended by nonphysician health care providers [χ^2^(6) = 59.983, *P* < .001], whereas physicians recommended surgery more frequently (52.6%).

Videos promoting traditional medicine were less likely [χ^2^(1) = 11.819, *P* = .001] to be rated as harmful (3.6%) compared with alternative medicine videos (22.7%). Videos with disagreeable comments were more likely [χ^2^(1) = 10.721, *P* = .001] to be perceived as harmful (33.3%) than those with agreeable comments (4.9%). There was a significant association between medical recommendations and context [χ^2^(2) = 21.778, *P* < .001]. Educational videos were more likely to include medical recommendations (37.7%) than anecdotal videos (11.4%), whereas commercial videos had the fewest medical recommendations overall (20.0%).

## Discussion

TikTok videos on acute knee injuries showed high viewer engagement but frequently provided incomplete or inaccurate medical information, with videos making medical recommendations having higher engagement whereas physicians make up a minority of creators. This study investigated the quality and engagement of TikTok videos related to knee injuries, and the findings paint a complex picture. Videos included in the data analysis that included medical recommendations consistently attracted more engagement and tended to be longer, which suggests that viewers are seeking out informative, guidance-oriented content. It is interesting to note that videos labeled subjectively satisfactory by independent reviewers were not only more engaging but also were clearer in their aims, were more relevant, and cited more sources than videos deemed of poor quality subjectively, which were often shorter and less reliable. Despite the high average view count (around 699,235 views per video), only a small fraction of these videos met high standards of educational quality as measured by the DISCERN criteria, pointing to a gap between what is popular and what is scientifically sound. Educational content was the largest category, mostly covering conservative treatment methods, exercises, and recovery tips. Although only 24.4% of these videos were made by professionals such as physicians, their content generally received higher quality scores. We also saw that profit-oriented accounts had greater numbers of followers and engagement scores, which could mean that commercial interests are influencing reach, potentially at the cost of content quality. Overall, our findings reveal a wide range of quality in knee injury content on TikTok, with a noticeable gap between reliable, high-quality health information and the popular content users are actually consuming.

The results presented within this study correspond to previous research showing persistent quality issues in social media health content.^9^, ^10^, ^11^ For instance, Kolade et al.^4^ (2023) identified similar inaccuracies in posts on platforms such as Instagram and YouTube (Alphabet), particularly for orthopaedic injuries such as ACL and meniscal tears. This suggests that social media often prioritizes accessibility and engagement over depth and accuracy, which can lead to misinformation. Gieg et al.^5^ (2023) showed that health literacy has a great impact on patient expectations and outcomes, especially in cases involving surgical interventions. The high engagement in TikTok videos with medical recommendations observed in our study supports this idea, implying that patients gravitate toward content that offers guidance, even if it is not always from a reliable source. Although the relationship with medical educational quality has been investigated previously,^12^^,^^13^ by applying the DISCERN criteria to TikTok videos, we brought a structured approach to evaluating information quality on a platform that typically favors shorter, visually engaging content. As such, TikTok poses both a unique challenge and an opportunity for health communication because the brevity of videos may limit depth, emphasizing the need for clear, reliable information from credible sources.

The findings in this investigation about misinformation and potential harm echo what other studies have found regarding social media health content.^12^, ^13^, ^14^, ^15^, ^16^ Nonprofessional creators offering medical advice can pose a risk to the general population, especially when recommendations are not grounded in evidence or with scientific backing. Previous studies of YouTube and Instagram found similar trends, where influencers without medical backgrounds frequently shared advice on orthopaedic issues without citing reliable sources.^17^, ^18^, ^19^, ^20^ Our results align with this given that nonprofessional creators were more likely to give medical advice, with 35.2% of nonphysician creators providing recommendations compared with only 17.5% of physicians. Additionally, nonprofessional creators often suggested conservative treatment (57.2%) over surgical options (37.1%), which may contribute to misunderstandings about treatment for more severe injuries and deter viewers from considering medically necessary procedures.

It is important to note that videos created by health care professionals generally scored higher in terms of quality, with physician-created videos scoring better across DISCERN categories such as clarity of aims (mean score, 3.91; 95% CI, 3.75-4.06; *P* < .001) and relevance (mean score, 3.20; 95% CI, 3.07-3.32; *P* < .001) than poor-quality videos created by nonprofessionals. However, health care professionals make up a limited proportion of social media creators on platforms such as TikTok, as observed in other studies,^19^^,^^20^ with only 24.4% of videos in our sample attributed to physicians. This pattern underscores the need for more evidence-based information and stronger professional engagement on TikTok to counterbalance the commercially driven and sometimes misleading content.

This study adds to existing literature by specifically focusing on TikTok as an emerging hub for health information on knee injuries. Unlike research on other platforms such as YouTube and Instagram, we address TikTok’s short-form, visually focused content, which comes with its own set of challenges and opportunities. By looking at engagement metrics alongside DISCERN quality scores, we highlight how certain video features, such as length, medical recommendations, and creator background, affect both engagement and perceived quality. Our study also sheds light on the balance between professional and nonprofessional content, as well as the influence of commercial creators. The high engagement with commercial content raises questions about motivations behind health information on TikTok and what this means for patient understanding. These findings emphasize the need for more professional involvement and evidence-based recommendations on social media to improve the accuracy of health information that reaches patients.

### Limitations

The DISCERN scoring system was adapted for this study; it was originally designed to evaluate written consumer health information and has not been formally validated for video-based content. Although DISCERN scoring has been adopted in previous peer-reviewed studies assessing health information on platforms such as TikTok and YouTube, its application should be considered an adaptation. Video selection methods were formulated by drawing on previous social media health information evaluation studies and judgment to ensure the selected videos were those being frequently accessed by the general public, but there is no validated selection tool for social media videos adjacent to a tool such as the Preferred Reporting Items for Systematic Reviews and Meta-analyses (PRISMA) tool for systematic reviews. Although social media platforms such as TikTok have internal algorithms that may change video results from user to user and thus bias data queries, we attempted to mitigate this by using a new TikTok account with no history, using incognito browsing, and thoroughly querying for the most viewed videos per search term. Using the DISCERN criteria may not fully capture quality on a platform such as TikTok, on which brevity and visuals often win out over depth. We also assessed “harmful” content and “agreeable” comments subjectively, which brings in potential bias because harm is open to interpretation and may not directly connect to actual patient outcomes. Additionally, we were unable to track the long-term effects of these videos on viewers’ health behaviors, so we do not know how content impacts patient decisions over time. Finally, we did not delve into the creators’ intentions, which could reveal whether misinformation is a byproduct of simplification for engagement or solely a lack of medical knowledge.

## Conclusions

Videos created by health care professionals, particularly physicians, scored higher in terms of educational quality but accounted for a small proportion of total content. In contrast, nonphysician creators frequently provided inaccurate or incomplete information. Despite this, videos with medical recommendations achieved higher engagement.

## Disclosures

All authors (L.M.T., J.J., N.K.B., L.K.) declare that they have no known competing financial interests or personal relationships that could have appeared to influence the work reported in this paper.
